# Stress Induced Polarization of Immune-Neuroendocrine Phenotypes in *Gallus gallus*

**DOI:** 10.1038/s41598-017-08733-0

**Published:** 2017-08-14

**Authors:** F. Nicolas Nazar, Inma Estevez, Silvia G. Correa, Raul H. Marin

**Affiliations:** 1 Instituto de Investigaciones Biológicas y Tecnológicas (IIByT, CONICET-UNC) e Instituto de Ciencia y Tecnología de los Alimentos, Facultad de Ciencias Exactas, Físicas y Naturales, Universidad Nacional de Córdoba, Cordoba, CP 5000 Argentina; 2 NEIKER-Tecnalia, Arkaute Agrifood Campus, Departamento de Producción Animal, Vitoria-Gasteiz E-01080 e IKERBASQUE, Basque Foundation for Science, Bilbao, Spain; 3 Centro de Investigaciones en Bioquímica Clínica e Inmunología (CIBICI, CONICET-UNC), Cordoba, CP 5000 Argentina

## Abstract

Immune-neuroendocrine phenotypes (INPs) stand for population subgroups differing in immune-neuroendocrine interactions. While mammalian INPs have been characterized thoroughly in rats and humans, avian INPs were only recently described in *Coturnix coturnix* (quail). To assess the scope of this biological phenomenon, herein we characterized INPs in *Gallus gallus* (a domestic hen strain submitted to a very long history of strong selective breeding pressure) and evaluated whether a social chronic stress challenge modulates the individuals’ interplay affecting the INP subsets and distribution. Evaluating plasmatic basal corticosterone, interferon-γ and interleukin-4 concentrations, innate/acquired leukocyte ratio, PHA-P skin-swelling and induced antibody responses, two opposite INP profiles were found: LEWIS-like (15% of the population) and FISCHER-like (16%) hens. After chronic stress, an increment of about 12% in each polarized INP frequency was found at expenses of a reduction in the number of birds with intermediate responses. Results show that polarized INPs are also a phenomenon occurring in hens. The observed inter-individual variation suggest that, even after a considerable selection process, the population is still well prepared to deal with a variety of immune-neuroendocrine challenges. Stress promoted disruptive effects, leading to a more balanced INPs distribution, which represents a new substrate for challenging situations.

## Introduction

Interactions among nervous, endocrine and immune systems have been vastly characterized in the last decades in vertebrates as well as in invertebrates^1–6^. In vertebrates, particularly in birds and mammals, physiological communicational paths and nexus at molecular, cellular and organismal levels evidence the interactive nature of what has been called immune-neuroendocrine (INE) system^2, 6–11^. This system nucleates integrated physiological responses in the organism, covering a wide spectrum of possibilities ranging from local inflammation to systemic stress responses. A concept that emerged in this area in relation to population studies is the existence of immune neuroendocrine phenotypes (INPs) that represent individuals neuro-hormonally and immunologically similar and different from another group within the same animal population^10, 12–14^. Each of the mentioned similar groups share common characteristics referred to: (i) neuroendocrine mediator concentrations, (ii) activity and expression of hormonal receptors and (iii) cytokine levels belonging to pro- or anti-inflammatory profiles.

The LEWIS/FISCHER paradigm is a well characterized and central model for INE as well as for classical immunological studies^12, 15–17^. In LEWIS rats with strong Th1-pro-inflammatory responses rich in mediators such as interferon (IFN)-γ and interleukin (IL)-12, basal levels of Corticosterone (CORT) are low. Instead, FISCHER rats show a low to moderate Th1-pro-inflammatory profile that correlates with high basal CORT levels and other associated complementary INE parameters. Broadening the scope of INPs in mammals, Elenkov *et al*. reported the existence of these phenotypes in humans in 2008^13^. They identified two subgroups in healthy human individuals with relatively low and high epinephrine outputs that had opposite innate cytokine profiles, providing a clear link with the LEWIS/FISCHER paradigm. In 2015 we described for the first time the existence of INPs in an avian species (*Coturnix coturnix*), thus extending the scope to which this notion may be considered and at the same time expanding the evolutionary interpretation of the phenomenon^10, 14^.

Animal husbandry and production in human related environments usually implies suboptimal group sizes, altered or forced social structures, manipulation routines, and other diverse situations reported to be stressful as well as important health challenges. Laboratory animals and domesticated poultry species are far from being an exception^18–21^. The stated situations require a series of responses from the INE mega-system in order to cope with the challenges pursuing the ultimate goal of maintaining homeostasis^22, 23^. Animals with opposite INPs have divergent stress responses as reported in studies with LEWIS and FISCHER rats submitted to different stressors^12, 13, 15–17^. Physiological insights of their INE interactions have shown different secretory activity of their hypothalamic cells. This congenital difference configures a number of parameters and biochemical responses, which together characterize the depicted INPs.

Research has centred its attention mostly on responses ranging from physiological to cellular or molecular aspects^1, 10, 24–27^. However, the analyses of the information considering eco-immunological backgrounds could lead to additional interpretations. As recently pointed out by Ashley and Demas^10^, much work is still needed to improve our understanding of the constraints and advantages created by the tripartite neuro-endocrine-immune interactions. In a previous study by Nazar *et al*., (2015b) we evaluated in laying hens the effects of a chronic social disruption, induced by changing the appearance of some of the penmates, on immune and stress response variables. Only hens housed in socially disrupted pens showed leukocyte changes after treatment that were consistent with a chronic stress. Hens in control non-disrupted pens remained unchanged and therefore stable throughout the whole study. Herein, we focused on the assessment of individual INE responses within the socially disrupted pens both prior and after chronic social stress disruptions. This study allowed us to answer three main questions: (1) Are the INPs described for *Coturnix coturnix* ^14^ also observed and similarly distributed in *Gallus gallus* (an avian species that has been submitted to a much longer history of intensive domestication process and directed selective breeding^28–33^)? And if so, (2) do the INPs remain within similar proportions (stable) prior and after the chronic stress disruption? And (3) are the same individuals conforming each of the INPs prior and after the chronic stress disruption?

Considering the theoretical framework described, we hypothesize that INE arrangements as those previously described in *Hommo sapiens sapiens, Rattus norvegicus* and *Coturnix coturnix* will generalize to the avian model *Gallus gallus*. On the other hand, we also expect these arrangements to be modulated by induced chronic stress, influencing the individual and population levels.

## Material and Methods

### Animals and rearing conditions

Newly hatched one-day-old Hy-line brown female chicks were obtained from Avigán Terralta hatchery and transported to the experimental poultry facility at the Neiker-Tecnalia research centre (Vitoria-Gasteiz, Spain). Immediately upon arrival, 300 chicks were randomly assigned to one of 12 pens and housed in groups of 10 or 40 birds, 6 pens per each group size. Birds were kept at the same density (8 birds/m^2^), management and housing/environmental conditions described elsewhere^34^. At two days of age, birds were individually identified with two white laminated paper tags placed on each wing side^35^. Before the laying period started, pens were also provided with proportional nest space. Birds were maintained in the described conditions and were stressed by an induced modification of the social group structure (see below) by altering the birds´ appearance (changing the colour of the head feathers, see below).

### Basal situation: all group with homogeneous appearance

Upon arrival, all birds within half of the pens were either maintained (100% unmarked) or their physical appearance was modified (100% marked) by placing a black mark with a non-toxic dye on the back of the head^34, 36^. Birds remained with the same initially assigned appearance until 34 wk. of age.

### Chronic social stress disruption: sequential changing of some group members’ appearance

Groups were altered by sequentially changing the appearance of 30, 50 and 70% of individuals when adults at 34, 38 and 44 wk. of age (1^st^, 2^nd^ and 3^rd^ change, respectively). These changes were accomplished by either randomly marking the birds’ head (for the initially unmarked homogeneous groups) or unmarking them (for the initially marked homogenous groups). The manipulation used is known to engender chronic stress responses in the group members^36, 37^ and thus representing a social stressor: a pre-established social dynamic mismatch and at the same time the reestablishment and adaptation to the new social structure^34, 38^. Corticosterone and haematological indicator (heterophil/lymphocyte ratio) were assessed to ensure the applied protocol was indeed inducing a chronic stress reaction (see Supplementary Fig. S1).

### INPs determination

INP characterizations through analyses of INE variables were performed within the same ontogenetic phase (fully grown and mature hens). Measurements were taken at 29 wk. of age (basal - prior to the beginning of social stress disruption) and at 46 wk. of age (post-chronic social stress disruption −2 wk. after the last alteration was applied). Six randomly chosen birds within each pen (treatment replica) were designated to analyse INE variables in order to determine their basal and post-chronic social stress INPs (same animals were analysed prior and after chronic social stress disruption). A total of 72 birds were studied.

Based on previous reports of INPs in mammals (*Rattus norvegicus* ^12, 15^ and *Homo sapiens sapiens* ^13^), and our previous data in birds (*Coturnix coturnix* ^14^), the following INE variables were evaluated: plasma CORT as representative of the hypothalamus-pituitary-adrenal axis; skin-swelling response to *Phytohemagglutinin-p* (PHA-P), antibody response against sheep red blood cells (SRBC) and leukocyte populations as INE effectors; and two different INE interplay mediators that favour opposite milieu polarization: IFN-γ (pro-inflammatory) and IL-4 (anti-inflammatory). These two mediators have also been described as the hallmarks of pro- and anti-inflammatory INE responses in *Gallus gallus* ^39–41^.

### Sampling procedure

The complete sampling procedure both in basal and post-chronic social stress disruption took 3 days, within a period of one week. To ensure a reliable CORT value, the extraction procedure took no longer than 80 s from the moment the animal was initially captured. On day 1, the brachial vein of the bird’s left wing was punctured in order to obtain 1 ml of EDTA-anticoagulated blood for smears and for quantifying basal concentration of plasmatic CORT, IFN-γ and IL-4. Plasma was obtained by blood centrifugation at 2,500 g during 15 min and it was immediately stored at −20 °C until further analyses. At the same time PHA-P skin-swelling response was induced (see below). Immediately after, birds were intraperitoneally injected with 0.5 ml of a 10% SRBC suspension in order to induce a humoral immune response (see below). One week later blood was from the right brachial vein for SRBC primary antibody response assessment.

### Determinations

#### Skin-swelling response to PHA-P

To determine birds *in-vivo* general proinflammatory potentiall^42^, the responses to PHA-P, a lectin from *Phaseolus vulgaris* (Sigma Chemical, St. Louis, MO), was measured in the wing web of each bird following the methods described elsewhere^42–45^. Briefly, on day 1, a 0.05 ml intradermal injection of a solution of 1 mg/ml PHA-P in phosphate saline buffer (PBS) was injected in the wing web of each bird. The dermal swelling response was measured as the percentage increase in wing web thickness at the injection site 24 h post-PHA-P injection (day 2). Measurements were recorded to the nearest 0.01 mm using a mechanic digital micrometre.

#### Innate/acquired (INN/ACQ) ratio

Leukocyte counts were performed in blood smears stained with May-Grünwald Giemsa. Differential counts of 100 white cells per blood smear were made^46, 47^. The INN/ACQ cell ratio was calculated using the following formula: INN/ACQ = (number of basophils + number of heterophils + number of monocytes)/(number of eosinophils + number of lymphocytes).

#### Antibody response against SRBC

To evaluate the induced humoral immune response, the antibody titer was assessed with a micro agglutination assay^43, 48, 49^. Briefly, one week after the intraperitoneal administration of the SRBC suspension we took blood samples and 20 μl of complement-inactivated plasma (through heating to 56 °C) was serially diluted in 20 μl of PBS. Next, 20 μl of a 2% suspension of SRBC in PBS was added to all wells. Microplates were incubated at 40 °C for 1 h, and hemagglutination of the plasma samples was compared to the blanks (PBS only) and negative controls (wells with no SRBC suspension). Antibody titters were reported as the Log2 of the highest dilution yielding significant agglutination.

#### Plasma CORT, IFN-γ and IL-4 determinations

Plasma CORT (pg/ml) was quantified using a validated specific CORT ELISA kit (ENZO Life Sciences - ADI-901-097)^50^. IFN-γ and IL-4 (pg/ml) were quantified using validated specie-specific ELISA kits (Uscn Life Science Inc., IFN-γ: E90049Ga; IL-4: E90077Ga). The reactivity with CORT was 100% with a sensitivity of 27.0 pg/ml detecting concentration ranging from 32 to 20.000 pg/ml. The cross reactivity of related steroids compound was: deoxycorticosterone 28.6%, progesterone 1.7%, testosterone 0.13%, tetrahydrocorticosterone 0.28%, aldosterone 0.18%, cortisol 0.046, pregnenolone and cortisone <0.03%. The reactivity with IL-4 was 100% with a sensitivity of 15.63 pg/ml detecting concentration ranging from 16 to 1.000 pg/ml. The reactivity with IFN-γ was 100% with a sensitivity of 15.63 pg/ml detecting concentration ranging from 16 to 1.000 pg/ml. No significant cross-reactivity or interference between the analysed cytokines and their analogues was observed. Intra- and inter-assay variability for each target molecule were 8.4% and 8.2% (corticosterone), 9% and 11% (IL-4 and IFN-γ). Determinations were done following the procedure specified by the manufacturer. All samples for each target molecule were assessed the same day and by duplicate.

### Statistical analyses

Correlation analysis between birds sampling order and every variable analysed were performed in order to ensure there was no influence of this procedure on the obtained results. An ANOVA on previous and post-chronic social stress data that included birds’ appearance (marked vs. unmarked) and GS (10 vs. 40) as a factors was also performed to rule out potential influences of those variables or their interactions on the results of the INE studied variables.

### Basal analyses

First, a cluster analysis was performed in order to explore the degree of similarity among the birds in the study on the bases of the analysed variables (factors). Particularly, with the aim of identifying the best partition of the birds, described by the best orthogonal linear combinations of the factors according to the least-squares criterion, a K-means analysis was performed. The following 6 standardized variables were used in the analysis: plasma CORT, IFN-γ and IL-4, skin-swelling response to PHA-P, antibody response against SRBC and INN/ACQ ratio. Based on the theoretical framework previously exposed, the clustering was performed with a K value of 3 (2 subgroups with representatives of potential extreme INPs and 1 intermediate subgroup). Euclidean metrics were used for computing the distance between points and cluster centres. To graphically represent the results of the clustering in a multivariate manner, a Principal Component Analysis (PCA) was used with the 6 clustering variables as explanatory variables. Birds were labelled with their categorization according to the cluster they belong to. Lastly, to fully characterise each cluster, a means comparison was also done with a univariate general linear mixed (glm) model assuming Gaussian error distribution with an identity-link function for each response variable. Cluster was used as a fixed effect and pen identity was included as a random effect. Differences between the three clusters on basal CORT, IFN-γ and IL-4 levels, skin-swelling response to PHA-P, antibody response against SRBC and INN/ACQ ratio were studied.

### Post-chronic social stress disruption analyses

Because it is expected that not all individuals will show the same stress response and therefore contributing differentially to the stress response at a population level, we used the following approaches:

- K-means analyses was performed with post-chronic social stress data (K = 3, Euclidean metric). PCA bi-plot was again used as a graphical strategy. To characterize the identity of each clustered subgroup post-chronic social stress, univariate general linear mixed model was used assuming normal distribution of the data, the 3 cluster subgroups as a fixed factor and pen and each animal identification as random factors. Differences between the cluster subgroups on basal CORT, IFN-γ and IL-4 levels, skin-swelling response to PHA-P, antibody response against SRBC and INN/ACQ ratio were studied again.
- Proportion tests were used to quantitatively characterize the change in the number of birds within each cluster obtained previous and post-chronic social stress disruption.

In every case, a *p*-value < 0.05 was considered to represent significant differences. All statistical analyses were performed through an ‘R’ (The R Foundation for Statistical Computing) user-friendly interface implemented in InfoStat^51^. Whenever significant main effects were detected, Fisher’s least significant difference (LSD) was used to compare the means. Cluster was performed using *kmeans* and models were fitted using the *lme* and *glm* R packages.

## Results

No correlations were found between sampling order and the analysed variables in neither basal conditions nor post-stress (p > 0.65 in all cases). Furthermore, no significant effects were found on the influence of hens´ appearance (head feathers marked vs. unmarked), group size (10 vs. 40 individuals) and their interaction on the studied INE variables (*p* > 0.47 in all cases). Consequently, sampling order, hens’ appearance and group size were not included as influencing factors in the subsequent analysis.

### Basal situation: all group with homogeneous appearance

**Figure 1:**
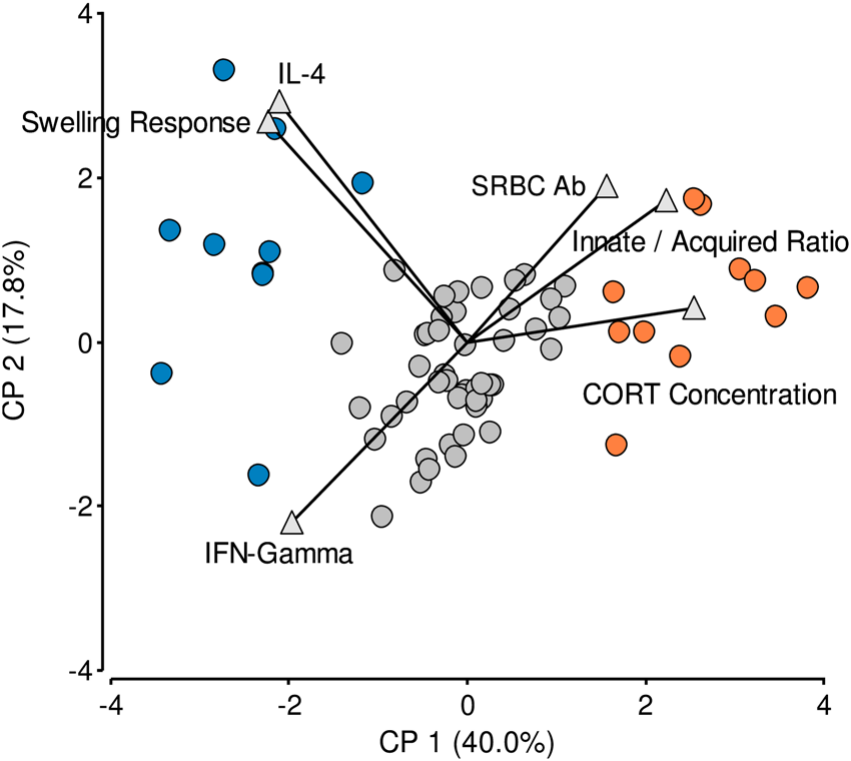
Exploration of data variability and clustering procedure. Principal component analysis bi-plot graphs. Each bird is represented by a dot and each explanatory and clustering variable used in the analysis is represented by triangle. Total distribution of the data categorized according to the birds’ assignation according to k-means clustering. Full blue dots and full orange dots represent extreme clusters with low and high basal CORT concentration respectively; full grey dots represent birds with intermediate configuration. The eigenvalues are shown in brackets next to each component. Number of birds in the study: 67.

K-means analysis distributed the birds in 3 main dissimilar groups. The largest group consisted of 46 birds and the other 2 of 11 and 10 birds. The principal component analysis bi-plot labelled with the cluster categorization indicates that the groups with lower number of hens were located in the extreme of the plotted multivariate space and the larger group in the centre (Fig. 1). Distances between dots in the bi-plot are also an indicator of the within group similarities (see Supplementary Table S5 for PCA statistical details). Coherently, the characterization of the INE variables also informed that the two smaller groups have opposite responses in all variables studied and that the larger group was less homogeneous, consisting of animals with intermediate or partially shared responses with the extreme groups.

**Figure 2:**
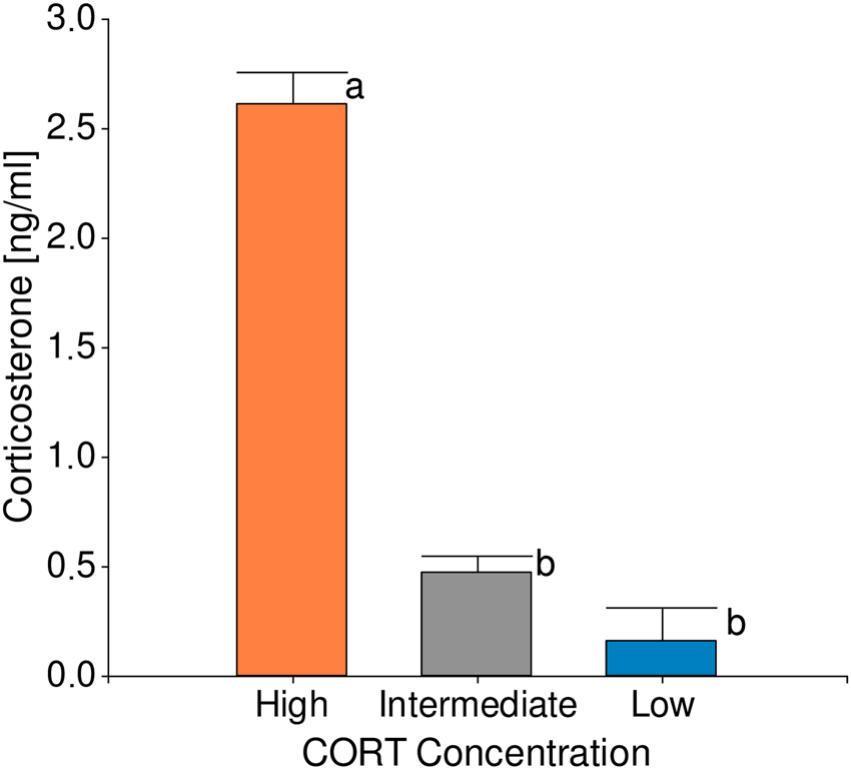
Divergent groups basal CORT concentration. Effect of clustering on basal CORT concentration. Low and High CORT birds where identified according to their hormonal concentration. Data are means ± SE. Different letters indicate significant (p < 0.05) differences between groups. Number of birds in the study = 67, number of birds per group Low = 10, High = 11, Intermediate = 46.

**Figure 3:**
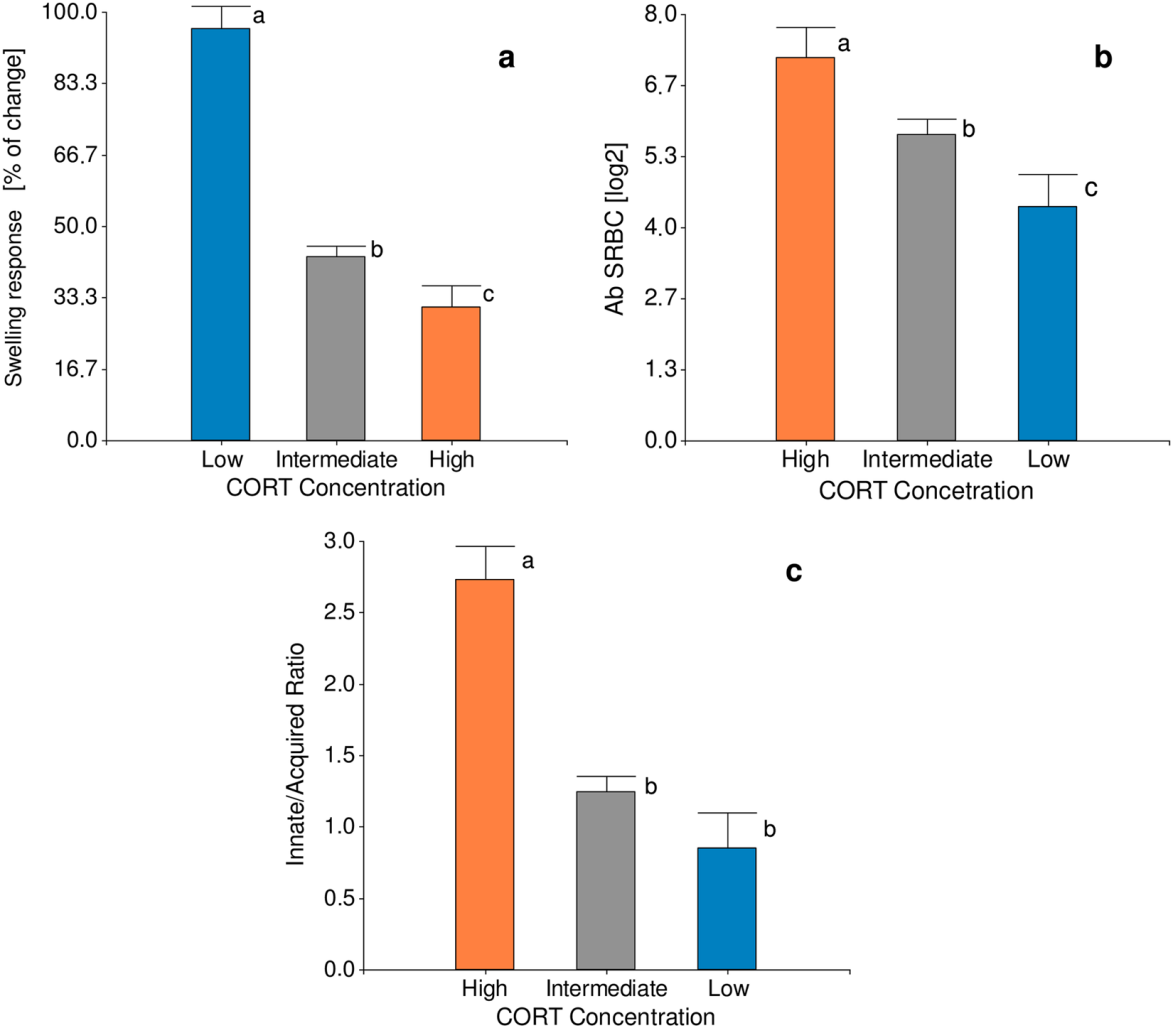
Immune effector analysis in divergent Low and High CORT groups. Effect of clustering on immune effectors is shown in (**a**) skin-swelling response to PHA-P, (**b**) antibody response against SRBC and (**c**) INN/ACQ. Data are means ± SE. Different letters indicate significant (*p* < 0.05) differences between groups. Number of birds per group Low = 10, High = 11, Intermediate = 46. INN/ACQ number was calculated using the following formula: INN/ACQ = (number of basophils + number of heterophils + number of monocytes)/(number of eosinophils + number of lymphocytes).

**Figure 4:**
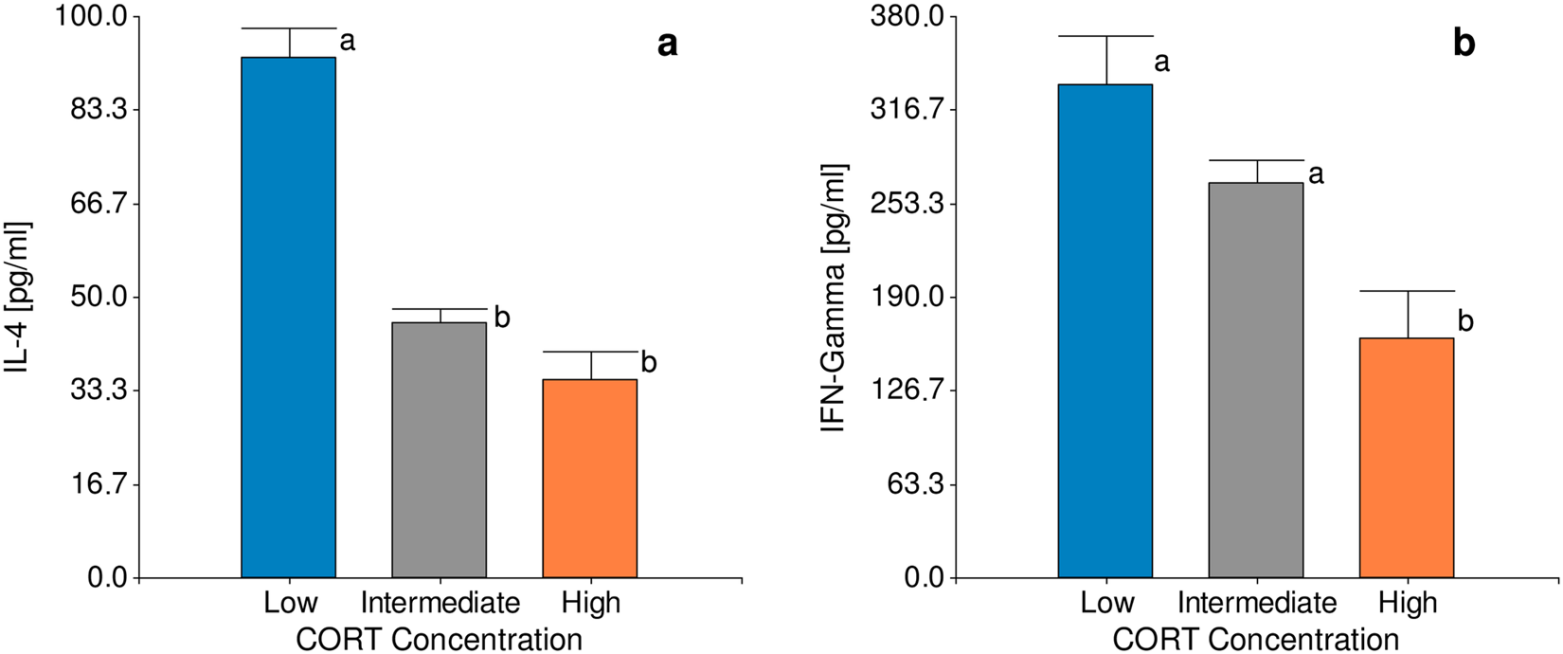
Analysis of molecular mediators in divergent Low and High CORT groups. Effect of clustering on the expression of (**a**) anti-inflammatory (IL-4) and (**b**) pro-inflammatory (IFN-γ) mediators. Data are adjusted means ± SE. Different letters indicate significant (p < 0.05) differences between groups. Number of birds per group: Low = 10, High = 11, Intermediate = 46.

Statistical evaluation of the INE variables after the cluster analysis showed that in the basal situation all measurements are also polarized, thus reinforcing the consistency of each cluster classified subgroup. Comparing the two extreme groups we found that they vary in their basal CORT concentration (*F*_2,51_ = 91.82, *p* *<* 0.001; Fig. 2). Hens with lowest CORT concentration showed higher skin-swelling responses to PHA-P (*F*_2,51_ = 46.39, *p* < 0.001; Fig. 3a), higher antibody response against SRBC (*F*_2,51_ = 5.69, *p* < 0.005; Fig. 3b), and lower INN/ACQ ratio (*F*_2,51_ = 19.28, *p* < 0.001; Fig. 3c) than their high CORT counterparts. To reveal if the clusters defined also exhibited a molecular substrate underlying the putative INE phenotypes we also evaluated the pro-inflammatory (IFN-γ) and anti-inflammatory (IL-4) cytokines. The analysis revealed that opposite groups differed in these mediator concentrations (Fig. 4). Animals with low basal CORT showed significantly higher levels of IFN-γ (*F*_2,51_ = 7.07, *p* < 0.01; Fig. 4a) and IL-4 (*F*_2,51_ = 38.61, *p = *0.001; Fig. 4b) in comparison with their high basal CORT counterparts. It is worth mentioning that although animals with low CORT levels have significantly elevated both pro and anti-inflammatory cytokine production, IFN-γ values had a four-fold increment showing the predominance of this mediator determining a particular milieu.

**Figure 5:**
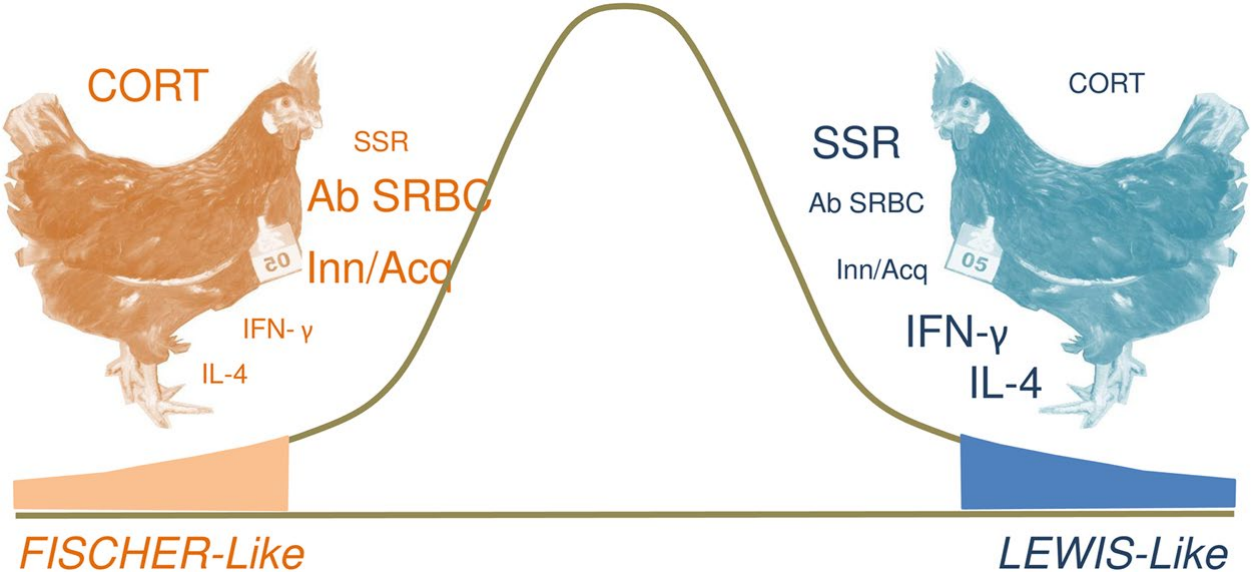
Schematic conceptual representation of INPs in *Gallus gallus*. The variables determining the existence of INPs are represented around each bird. The size of the variable indicates if the animals show high or low response in each of the parameters. SSR: skin-swelling response to PHA-P; Ab SRBC: antibody response against SRBC; Inn/Acq: innate/acquired leukocyte ratio; level of expression of pro-inflammatory (IFN-γ) and anti-inflammatory (IL-4) mediators. ‘FISCHER-like’ hens with high CORT concentration also manifest high Innate/Acquired and Ab SRBC, but low SSR, IFN-γ and IL-4 levels. ‘LEWIS-like’ counterparts have low CORT as well as low Innate/Acquired and AB SRBC responses, but high SSR, as well as INE mediators (IFN-γ and IL-4) response.

Because of the physiological arrangements described, consisting of clusters of animals showing polarized INE responses comparable to the LEWIS/FISCHER paradigm^10, 12, 14, 15^ (Fig. 5) the two groups formerly defined are herein named ‘LEWIS-like’ or ‘FISCHER-like’ (animals with the lowest or the highest CORT concentration respectively and the other INE variables polarized accordingly).

### Post-chronic social stress disruption: sequential changing of the group members’ appearance

Similar procedures and analyses of data described for the basal situation were performed to evaluate individual and population immune-neuroendocrine responses post-chronic social stress disruption.

**Figure 6:**
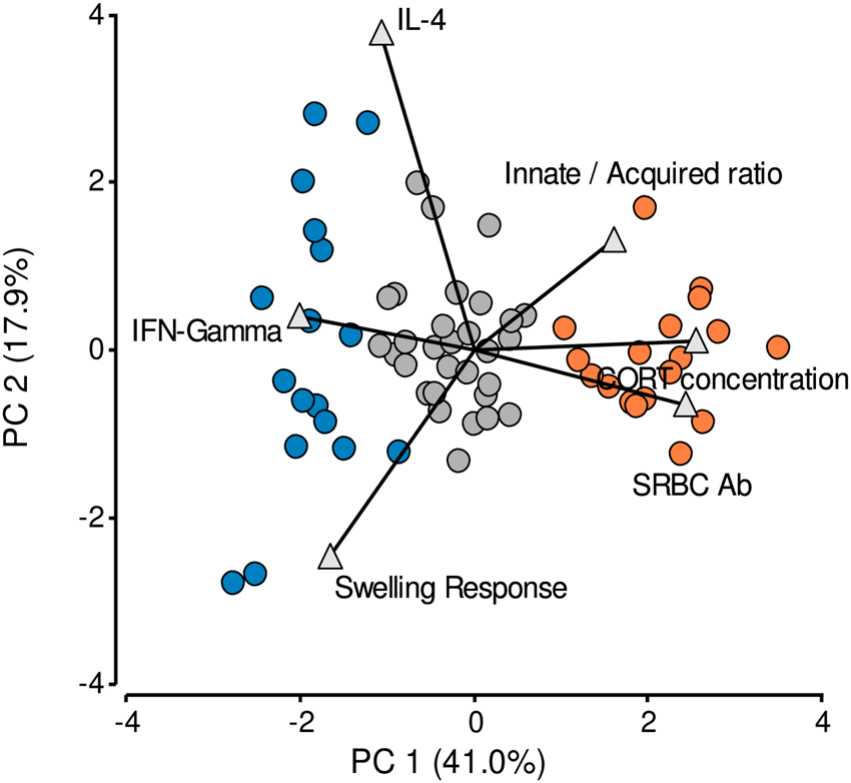
Exploration of data variability and clustering procedure post- chronic social stress. Principal component analysis bi-plot graph. Each bird is represented by a dot and each explanatory and clustering variable used in the analysis is represented by triangle. Total distribution of the data categorized according to the birds’ INP after stress disruption. Full blue dots and full orange dots represent extreme LEWIS- and FISCHER-like birds respectively; full grey dots represent birds with intermediate configuration. The eigenvalues are shown in brackets next to each component. Number of birds in the study: 65. Number of birds per group: LEWIS-Like = 17, FISCHER-Like = 18, Intermediate = 30.

**Figure 7:**
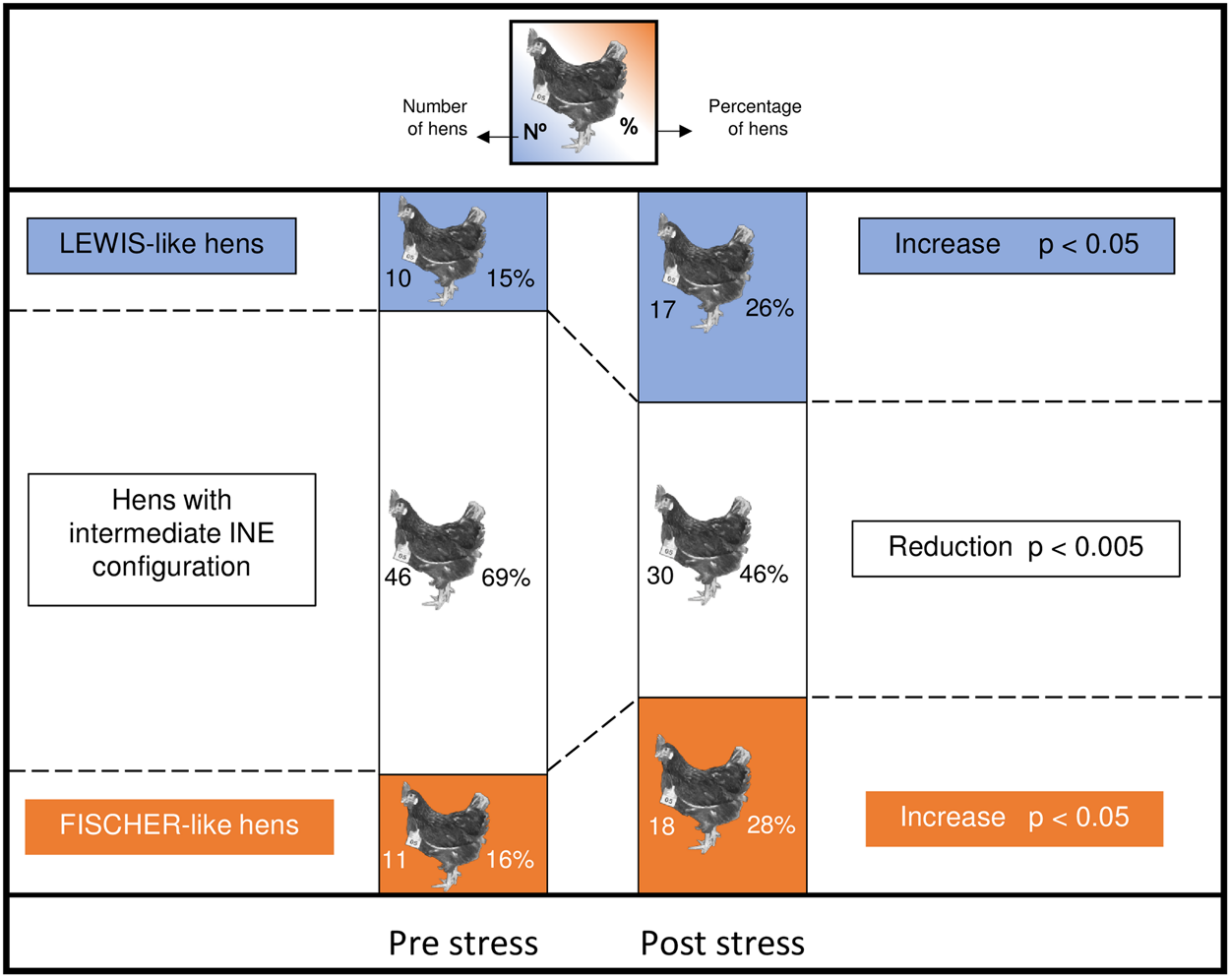
Proportion analysis of stress induced effects. The studied population is schematized previous and post chronic social stress impairment, as cumulative bars. In each bar the different INE configurations are represented in sections with blue (LEWIS-like), white (intermediate) or orange (FISCHER-like) stuffing. Information regarding absolute number and percentage of birds is also given. The final statistical and biological effect of the stress impairment is shown together with p-values. In every case, a p-value < 0.05 was considered to represent significant differences. Total number of animals previous stress = 67, total number of animals post stress = 65.

K-means analysis shows again 3 differentially-sized groups. Nevertheless, post-chronic social stress the size of the groups appeared more balanced than prior stress. The largest group in this situation consists of 30 birds and the other 2 of 18 and 17 birds. Figure 6 shows the representation of individual responses obtained in the multivariate space. The PCA bi-plot labelled with the cluster categorization again localises the two minor groups in the extremes of the plotted multivariate space and the largest one in the centre. INE arrangements found (see Supplementary Figs S2, S3 and S4 and Supplementary Table S6) and numerical analyses (Fig. 7) indicate that post-chronic social stress, 18 birds express an extreme INE association equivalent to a FISCHER-like configuration and 17 birds express the opposite configuration of the INE variables analysed, comparable to a LEWIS-like INP. The remaining 30 birds expressed an intermediate INE configuration (Figs 6 and 7).

The subgroups with the extreme INE configurations were mostly composed with the same individuals already categorized prior to stress. The 86% (18 out of 21) of the birds remained showing the same profile post-chronic social stress disruption. However, other situations were also observed within the population. A wide percentage of birds showing intermediate profile (40%) did switch its profile towards one or the other of the two extreme INP. Proportion analyses showed (Fig. 7) that the number of birds expressing FISCHER-like and LEWIS-like configuration were respectively increased from 11 to 18 birds (*p* < 0.05) and from 10 to 17 birds (*p* < 0.05) post-chronic social stress. The described increments are consistent with the reduction found in the number of birds showing an intermediate configuration post-chronic social stress (reduced from 46 to 30 individuals, *p* < 0.005). Moreover, the percentage of birds maintaining an extreme INE configuration post-chronic social stress (86%) was significantly higher than the percentage of birds (60%) conserving their intermediate configuration (*p* < 0.01). The latest implies that the 40% of those birds initially expressing an intermediate configuration polarized their INE interplay towards an extreme INP post-chronic social stress.

## Discussion

*Gallus gallus* (the domestic chicken) is an excellent avian model involved in most avian research immunology and physiology due to its economic importance^6, 27, 39, 52–54^. In the present study, we explored and characterized the INPs’ distribution in a chicken laying strain. We also characterized INE interactions post-chronic social stress in order to reveal the effects of the challenging situation in the described interplay. In particular, we analysed whether a chronic social stress disturbance can modulate the INE interplay of each individual consequently affecting the INPs’ proportions and distributions at a population level. As previously mentioned, the reported data may have diverse interpretations regarding plasticity, polarization, and other concepts of eco-immunological and physiological importance.

Our results shows that the INP physiological arrangement previously described for *Coturnix coturnix* ^14^ are also observed in *Gallus gallus* providing further consistency to the phenomenon in focus. INPs have a physiological substrate underlying the arrangement of each variable measured. LEWIS-like birds show in basal situation higher PHA response, a marker of the individual general proinflammatory potential^42^, linked to a lower humoral immune response to the T-dependent antigen SRBC^39^. According to previous reports, adrenocorticotropic hormone and CORT are immunosuppressive and hypothalamus-pituitary-adrenal axis activity generally reduces lymphocyte and increases heterophil numbers ^6, 55–57^. This phenomenon could also explain the different distribution of leukocyte subpopulation found in the two extreme CORT groups. Findings imply that animals differing in their basal CORT levels also show dissimilar and opposite INE associated responses.

The observed generalization of the INPs in *Coturnix coturnix* to a *Gallus gallus* laying strain also leads to interrogations related to the potential relationship between domestication and the expression of INPs. Up to this point, these phenotypes have been described in *homo sapiens sapiens* ^13^ or in other species (birds or mammals)^14, 15^ that during the last decades have been living in environments highly influenced by humans. Moreover, their development has taken place under direct human manipulation through many generations of selective pressure with either scientific or commercial/productive purposes^6, 58–60^. In this context, the expression of different phenotypical arrays within an avian population could represent a strategy to keep the higher diversity of INE responses in human-related environments.

Relations between INE components and behaviour have already been studied showing a strong interaction between them^10, 23, 61–63^. Behavioural changes, such as induced flight-or-fight responses or social defeat, resulted in immune modulations and simultaneous changes in endocrine profiles^10^. Ritschoff *et al*.^61^ demonstrated that a territorial intrusion induced upregulation in NF-kappa-β (the key transcription factor that triggers pro-inflammatory responses) in different species (*Mus musculus*, *Gasterosteus aculeatus* and *Apis mellifera*). Thus, the different INPs found could partially or in full be a reflection of the environment within pens during development (social interactions established between hens); however, further studies are needed to confirm that hypothesis.

It is worth mentioning that both basal and post-chronic social stress exposure all animals were deemed healthy (free from helminthic parasite indicators in the faeces, blood smears with no indications of infections, and plumage and footpath condition suggesting a good welfare status). From the eco-immunological perspective, INPs diversity could be interpreted considering the ‘dilution effect’, which relates diversity with the success of parasites in a particular community associating diverse host communities with lower levels of parasitism^64–66^. The expression of different INPs in peer birds within the same population could be considered an application of the community original hypothesis mentioned above. For a given population of animals, to hold a diversity of responses, amplifies the potential collection of challenging organisms they can deal with. Thereby, the observed INE diversity would function as the bases of the ‘dilution effect’.

Regarding post-stress stability of the INPs proportions at the population level, the larger number of birds that changed their phenotype post-chronic stress belonged to the intermediate INP configuration. Complementary analyses informed that this flux was not polarized towards one profile selectively; on the contrary, it was balanced between both extreme INPs arrangements described. Plasticity is a central concept in biological sciences and has been defined as the ability of a certain genotype to produce distinct phenotypes when exposed to different environments throughout its ontogeny^67–71^. Herein the social stressor may have determined a different environment for birds compared to the basal situation in which they were reared (undisturbed condition). Taking into consideration that the same individuals were sampled, the genotype could be considered as a component that remained stable^67, 71^. Plasticity would have allowed the fluxes from intermediate to extreme configurations and vice versa that were observed in the INPs distribution post chronic stress. In this context, the major flux observed from intermediate to extreme configurations could be interpreted as a response to the social chronic stress disruption. Nevertheless, it is worth emphasizing that the polarization is not complete. In particular, about 60% of the initial subgroup still remains with an intermediate INE configuration. This new distribution leads to a different and new configuration for future challenges demanding plastic responses from the INE interplay and, at the same time, still shows a potential variability of responses. However further research is needed to evaluate the last hypothesis.

Studies in ecology and evolution lead Cohen *et al*. to propose in 2012 ‘physiological regulated networks’ (PRN) as the body of molecules and regulatory relationships that maintain and adjust homeostasis and facilitate performance at the whole-organism level. In these networks, single molecules defined as ‘integrators’ have the crucial role of synthesizing internal or external information thereby determining multiple aspects of PRN functioning. The findings of our study could also be discussed within the theoretical context of PRN^70, 72^. One of the key functions of PRN is to facilitate transitions among phenotype states, which is consistent with the observed INE adjustments from the basal to the post-chronic social stress state. The different variables selected to determine INPs would represent part of the scaffold in which external challenges are physiologically integrated and solved. At the same time, not every bird responded to challenging situations with a phenotypic transition (some of them retained their previous INP). This was particularly evident in individuals with extreme phenotypes in basal conditions that in most of the cases remained with extreme configurations post-chronic social stress. Different scenarios should be considered at the individual level regarding PRN background^70–72^. Firstly, each phenotype could represent a potential ‘state’ of the PRN^70^. These phenotypes coexist as ‘different states’ to give appropriate responses to the potential challenges presented. Secondly, focusing on the birds that changed their INP configuration post-chronic social stress exposure, the different INPs could be considered as distinct PRN states equally valid for an individual to adjust the organism physiology to environmental demands based on the possibilities that individual plasticity offers. On the other hand, we believe that the fact that some individuals retained extreme INP configurations does not invalidate the concept of plasticity but reinforces the idea that biological phenomena rarely happen in an ‘all or nothing’ manner.

Lastly, focusing the attention in the phenomenon at a population level, again two different scenarios could be described. In basal conditions 16% of the population expressed a Th1 biased response, 16% an opposite Th2 biased response and 58% an intermediate response. Post-chronic social stress the extreme percentages increased reaching 29% each at expenses of a loss in the intermediate subgroup of animals (final 32%). A remarkable positive aspect of both circumstances is the conserved variability intra-population (with the associated advantages previously mentioned). But, and very important in this case, the change in the frequencies post-chronic social stress led to a more balanced proportion of the three INPs. In the case of an eventual situation implying a significant reduction in the overall population, (e.g.: a bottle-neck or founding event)^73–77^ the chances of representation of each INE configuration could be higher when the substrate is a more balanced population. Again, the fact that biological phenomena do not occur at ‘all or nothing’ gains coherence and empirical consistency.

INPs observed in mammals and quail seems a more general phenomenon that also appears in an avian species with a much greater commercial relevance and, more importantly, that has been intensely manipulated through a long history of selective breeding. Findings suggest that at a population level, hens are prepared to deal with a wide spectrum of INE challenges. After chronic stress exposure, an increase in the number of birds showing extremes INP frequencies was found at the expense of a reduction in the number of hens showing intermediate responses. Observations suggest a stress disruptive effect on INPs distribution. The increased frequency in polarized INPs induced by chronic stress leads to a more balanced INPs distribution, which would be the new substrate for an eventual challenge demanding an INE response.

### Ethics approval

Animal care was provided in adherence with Institutional animal Care and Use Committee guidelines. The experiment was approved by the ethical committee at Neiker-Tecnalia in compliance with the Spanish legislation regarding the use of animals for experimental and other scientific purposes (Real Decreto 1201/2005).

All the data on which the conclusions of the paper are based are presented in the paper. Nevertheless, datasets used and/or analysed during the current study available from the corresponding author on reasonable request.

## Electronic supplementary material

Supplementary information
