# Supplementary material for: Stress Induced Polarization of Immune-Neuroendocrine Phenotypes in Gallus gallus
Source: Sci Rep. 2017 Aug 14;7:8102. doi: 10.1038/s41598-017-08733-0 (PMC5556001; doi:10.1038/s41598-017-08733-0)
Supplement: Supplementary file 1 — Supplementary information [file 41598_2017_8733_MOESM1_ESM.pdf]

Stress Induced Polarization of Immune-Neuroendocrine Phenotypes in *Gallus gallus*.

Authors.

F. Nicolas Nazar*, Inma Estevez, Silvia G. Correa and Raul H. Marin.

Supplementary information.

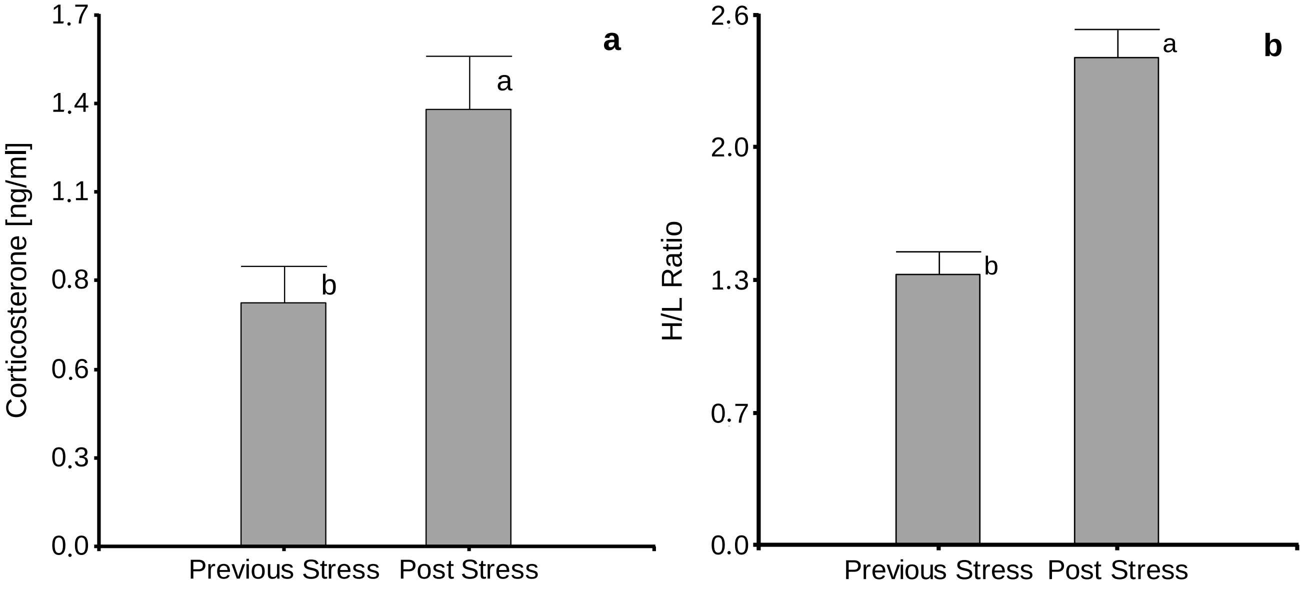

Supplementary Fig. S1. Effect of social chronic stress challenge on (a) Corticosterone concentration and (b) Heterophil/Lymphocyte (H/L) ratio. Different letters indicate significant ($p < 0.05$) differences between groups. Data are means ± SE. Number of birds per group: Previous Stress = 67, Post-Stress = 65. H/L ratio was calculated using the following formula: H/L = number of heterophils / number of lymphocytes.

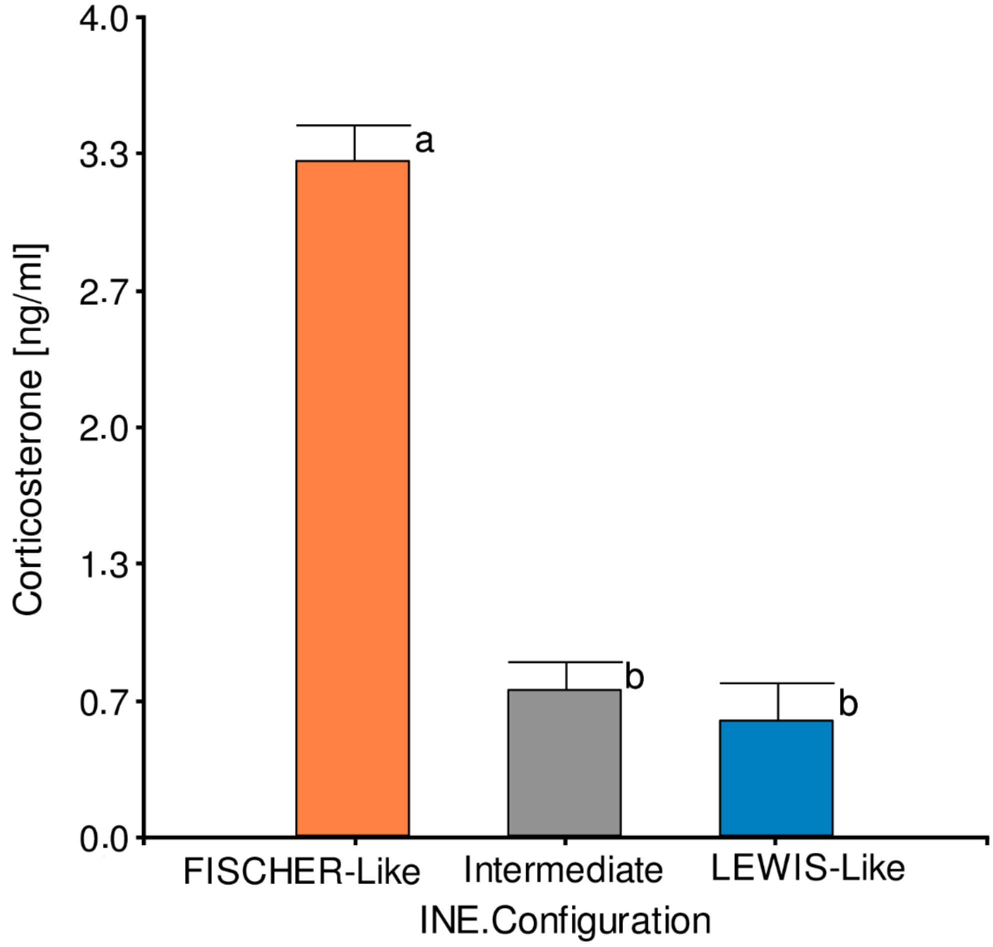

Supplementary Fig. S2. Intermediate, LEWIS-Like, FISCHER-Like groups' basal CORT concentration.

Effect of post stress clustering on basal CORT concentration. Low and High CORT birds where identified according to their hormonal concentration. Data are means ± SE. Different letters indicate significant ($p < 0.05$) differences between groups. Number of birds in the study = 65; number of birds per group: Low CORT = 17, High CORT = 18, Intermediate = 30.

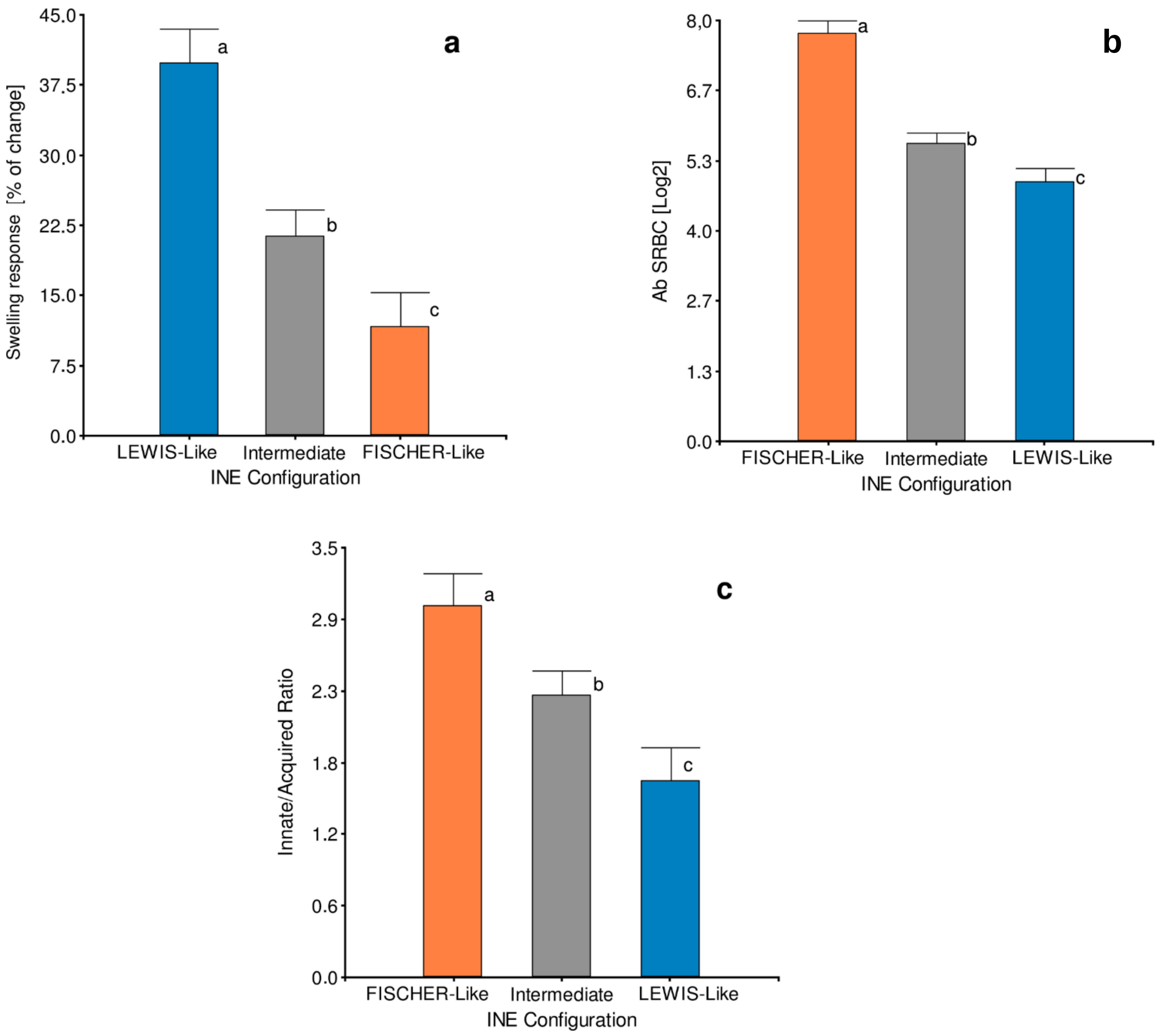

Supplementary Fig. S3. Immune effector analysis in Intermediate, LEWIS-Like, FISCHER-Like groups.

Effect of post stress clustering on immune effectors is shown in (a) swelling response to PHA-P, (b) antibody response against SRBC and (c) Innate/Acquired ratio. Different letters indicate significant ($p < 0.05$) differences between groups. Data are means ± SE. Number of birds per group: LEWIS-Like = 17, FISCHER-Like = 18, Intermediate = 30. INN/ACQ ratio was calculated using the following formula: INN/ACQ = (number of basophils + number of heterophils + number of monocytes) / (number of eosinophils + number of lymphocytes).

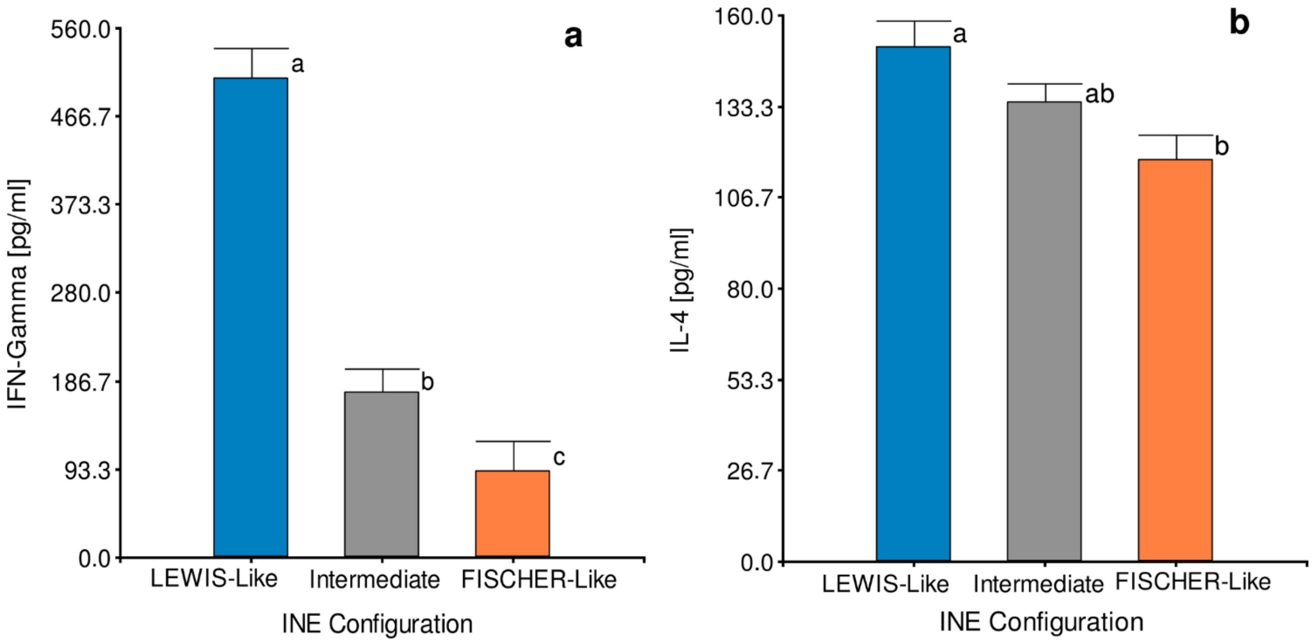

Supplementary Fig. S4. Analysis of molecular mediators in Intermediate, LEWIS-Like, FISCHER-Like groups.

Effect of post stress clustering on the expression of (a) anti-inflammatory IL-4 and (b) pro-inflammatory IFN-γ mediators. Data are adjusted means ± SE. Different letters indicate significant ($p < 0.05$) differences between groups. Number of birds per group: LEWIS-Like = 17, FISCHER-Like = 18, Intermediate = 30.

a

Principal component	Eigenvalue	Cumulative EV
**1**	0.4	0.4
**2**	0.18	0.58
**3**	0.14	0.72
**4**	0.12	0.83
**5**	0.09	0.93
**6**	0.07	1

b

Variable	Eigenvector 1	Eigenvector 2
**Corticosterone concentration**	0.49	0.08
**Swelling response**	-0.43	0.51
**Ab against SRBC**	0.30	0.36
**Innate / Acquired ratio**	0.43	0.33
**IL-4**	-0.41	0.56
**IFN-γ**	-0.38	-0.42

Supplementary Table. S5. Principal component analyses before social chronic stress disruption. Eigenvalues (a) and eigenvectors (b) for Principal Component Analyses used to graphically represent the results of the clustering in a multivariate manner, using the 6 clustering variables as explanatory variables.

a

**Principal component**	**Eigenvalue**	**Cumulative EV**
**1**	0.41	0.41
**2**	0.18	0.59
**3**	0.14	0.73
**4**	0.12	0.85
**5**	0.09	0.94
**6**	0.06	1

b

**Variable**	**Eigenvector 1**	**Eigenvector 2**
**Corticosterone concentration**	0.53	0.02
**Swelling response**	-0.35	0.52
**Ab against SRBC**	0.51	-0.14
**Innate / Acquired ratio**	0.33	0.27
**IL-4**	-0.23	0.79
**IFN-γ**	-0.42	0.08

Supplementary Table. S6. Principal component analyses after social chronic stress disruption. Eigenvalues (a) and eigenvectors (b) for Principal Component Analyses used to graphically represent the results of the clustering in a multivariate manner, using the 6 clustering variables as explanatory variables.
